# Evaluation of the Insecticidal Potential of *Heterotheca inuloides* Acetonic and Methanolic Extracts against *Spodoptera frugiperda* and Their Ecotoxicological Effect on *Poecilia reticulata*

**DOI:** 10.3390/plants12203555

**Published:** 2023-10-13

**Authors:** Karla Hernández-Caracheo, Lina Guerrero-López, Benjamín Rodríguez-Sánchez, Enrique Rodríguez-Núñez, José Luis Rodríguez-Chávez, Guillermo Delgado-Lamas, Juan Campos-Guillén, Aldo Amaro-Reyes, María del Carmen Monroy-Dosta, Carlos Eduardo Zavala-Gómez, Ricardo Chaparro-Sánchez, José Alberto Rodríguez-Morales, Víctor Pérez-Moreno, Miguel Angel Ramos-López

**Affiliations:** 1 Faculty of Chemistry, Autonomous University of Queretaro, Queretaro 76010, Mexicolinaguerrerolopez@gmail.com (L.G.-L.); aldo.amaro@uaq.edu.mx (A.A.-R.); ezavala2@gmail.com (C.E.Z.-G.); victorperezmoreno@yahoo.com.mx (V.P.-M.); 2 Ceickor University Center, Bernal Highway, Access to Ezequiel Montes Montes Km. 3, Los Benitos, CP., Queretaro 76299, Mexico; 3 Institute of Chemistry, National Autonomous University of Mexico, Mexico City 04510, Mexico; 4 Man and His Environment Department, Metropolitan Autonomous University Xochimilco Unit, Calzada del Hueso 1100, Coyoacan, Mexico City 04960, Mexico; 5 Faculty of Informatics, Autonomous University of Queretaro, Av. de las Ciencias s/n, Juriquilla 76101, Mexico; 6 Faculty of Engineering, Autonomous University of Queretaro, Queretaro 76010, Mexico; jose.alberto.rodriguez@uaq.mx

**Keywords:** *Spodoptera frugiperda*, *Poecilia reticulata*, ecotoxicologic, insecticide, botanical extract

## Abstract

For the management of *Spodoptera frugiperda*, botanical extracts have been used to reduce the environmental impacts of synthetic chemical pesticides. In the present investigation, the insecticidal activity of the acetonic and methanolic extracts of *Heterotheca inuloides* (Asteraceae) and of the main compound 7-hydroxy-3,4-dihydrocadalene on this pest as well as its ecotoxicological effect on *Poecilia reticulata* were evaluated. A greater insecticidal response was obtained from the acetonic extracts than from the methanolic extracts, with LC_50_ values of 730.4 ppm and 711.7 ppm for samples 1 and 2, respectively. Similarly, there was a lethal effect on 50% of the *P. reticulata* population at low concentrations in the acetonic extract compared to the methanolic extract. The sesquiterpene 7-hydroxy-3,4-dihydrocadalene has greater insecticidal activity by presenting an LC_50_ of 44.36 ppm; however, it is classified as moderately toxic for guppy fish.

## 1. Introduction

*Spodoptera frugiperda* is one of the main pests affecting corn (*Zea mays*; Poaceae) [1]. Various methods have been implemented to control this pest, including the use of synthetic chemicals such as carbamates, organophosphates and coumarins, due to their effectiveness, speed and broad spectrum [2]. Unfortunately, the use of these highly stable synthetic compounds not only causes a toxicological effect on pest insects but also generates resistance in target organisms, contamination of soil and water, and damage to human health, such as tumors, cancer and even death [3,4]. Different methods of controlling *S. frugiperda* with less environmental impact have been proposed, such as botanical management, which uses plant extracts to combat this insect pest [5]. Among the plant species that have been successfully used to control this pest are species of the families Lamiaceae, Euphorbiaceae, Rosaceae, and Asteraceae [6,7].

The use of botanical extracts is possible due to the synthesis of secondary metabolites in plant tissues that have a defensive effect against insects, including repellent activity and feeding, growth and oviposition inhibition, as well as a toxic effect, including interference in communication and sterilization [5]. Other characteristics of these extracts are their easy degradability, their selectivity, and their friendly interaction with the environment, so the use of plant extracts as botanical management has become a potential alternative for the management of *S. frugiperda* [8].

*Heterotheca inuloides* (Asteraceae), is a plant endemic of Mexico. In the wild, it is most commonly found in temperate pine-oak woodlands. However, it also grows in xerophytic scrub and semi-arid regions of Mexico. It is used in traditional Mexican medicine for the treatment of injuries, bruises and conditions associated with inflammatory processes. It is also reported to be used for the treatment of gastrointestinal disorders, circulatory ailments and respiratory diseases. Additionally, extracts and compounds isolated from the plant have shown different biological activities such as anti-inflammatory, analgesic, antioxidant, antimicrobial, chelating, and nematicidal activities. To date, more than 140 compounds have been isolated from its different extracts. The main secondary metabolites that have been reported in *H. inuloides* include groups of terpenes, fhytosterols and flavonoids [9,10].

The genus *Heterotheca* has reported acaricidal activity against *Varroa jacobsoni*, as well as larvicidal activity against *Spodoptera litura* and *Pseudoplusia includens* [10,11]. Botanical pesticides can be effective against pest insects; however, some of them can affect nontarget species, causing damage to the environment, so it is necessary to carry out studies that provide information on the possible biological impacts of these products. In this sense, toxicity tests are an appropriate tool that provides information on possible effects on human health and the environment by evaluating the contaminant of interest under controlled conditions on bioindicator species such as *Poecilia reticulata* [12,13,14,15]. The compound 7-hydroxy-3,4-dihydrocadalene has been isolated from the aerial parts of *H. inuloides* an LC_50_ of 45.47 µM in *Artemia salina* [9,16]. For this reason, the aim of this work was to evaluate the insecticidal activity of acetonic and methanolic extracts from the aerial parts of *H. inuloides* and also 7-hydroxy-3,4-dihydrocadalene against *S. frugiperda*, as well as its toxicological activity on *P. reticula.*

## 2. Results

### 2.1. Insecticidal Activity of the Acetonic and Methanolic Extracts of Heterotheca inuloides against Spodoptera frugiperda

In the insecticidal evaluation of the acetonic extract of sample 1, it was observed that for larval mortality, the concentrations of 5000, 2000 ppm and 1000 ppm showed significant responses with respect to the control, obtaining rates of 90, 70 and 85%, respectively. There was no significant difference in pupal mortality, while for cumulative mortality, all concentrations elicited significant responses starting at 500 ppm. The larval LC_50_ was 730.4 ppm, and the cumulative LC_50_ was 401.5 ppm. In the evaluation carried out with the acetonic extract of sample 2, it was observed that the 600 and 1000 ppm treatments showed significant responses in larval mortality, with rates of 40 and 70%, respectively. In the case of pupal mortality, no significant response was recorded. In contrast, at the concentration of 1000 ppm, a significant cumulative mortality was observed, with only 30% of the initial organisms surviving. The larval LC_50_ was 711.7 ppm, and the cumulative LC_50_ was 609.8 ppm (Table 1).

The bioassay with the methanolic extract of sample 1 showed that the concentration of 5000 ppm resulted in a significant larval mortality response, with a rate of 85%. For pupal mortality, significant responses were observed at 1000 and 2000 ppm, with a mortality rate of 35% for both concentrations.

In the case of cumulative mortality, significant responses were obtained at 1000, 2000 and 5000 ppm, with mortality rates of 70, 75 and 85%, respectively. The larval LC_50_ of the sample was 2302 ppm, and cumulative LC_50_ was 1033.8 ppm. The bioassay carried out with the methanolic extract of sample 2, the concentration of 1000 ppm showed a significant larval mortality, with a rate of 60%. In the case of pupal mortality, no concentration yielded a significant response. For cumulative mortality, concentrations of 600 and 1000 ppm showed significant responses, with rates of 55 and 65%, respectively. The larval LC_50_ was 781.3 ppm, and cumulative LC_50_ was 681.3 ppm (Table 2).

### 2.2. Ecotoxicological Activity of the Acetonic and Methanolic Extracts of Heterotheca inuloides against Poecilia reticulata

Once the biological activity of the acetonic and methanolic extracts of *H. inuloides* on *S. frugiperda* was observed, its toxicological effect on fingerlings, adult males and females of *P. reticulata* was determined (Table 3). At the concentrations of 500 ppm in fingerlings and 250 ppm in adult males and females, a mortality rate of 100% at 6 h was observed for all bioassays.

In the case of the methanolic extract (Table 4), it was observed that at the concentration of 250 ppm, there was a cumulative mortality of 100% in fingerlings after 72 h, with an LC_50_ of 187.16 ppm and a TL_50_ of 23.09 h. For the bioassays with male adults, the 500 ppm concentration was the only treatment that showed 100% mortality as an cumulative effect during the 96 h of exposure, and its LC_50_ was 223.97 ppm with a TL_50_ of 1.32 h. In contrast, the tests carried out in female adults showed a similar effect as in male adults because, at the concentration of 500 ppm, there was a cumulative mortality of 100% with an LC_50_ value of 153.74 ppm and a TL_50_ of 0.5 h.

### 2.3. Identification of 7-Hydroxy-3,4-dihydrocadalene

**Figure 1:**
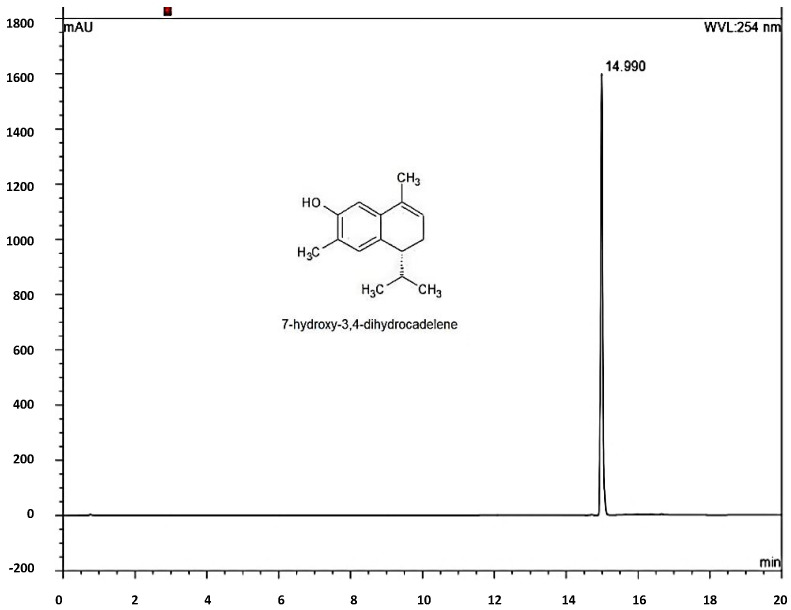
Chromatogram of 7-hydroxy-3,4-dihydrocadalene.

**Figure 2:**
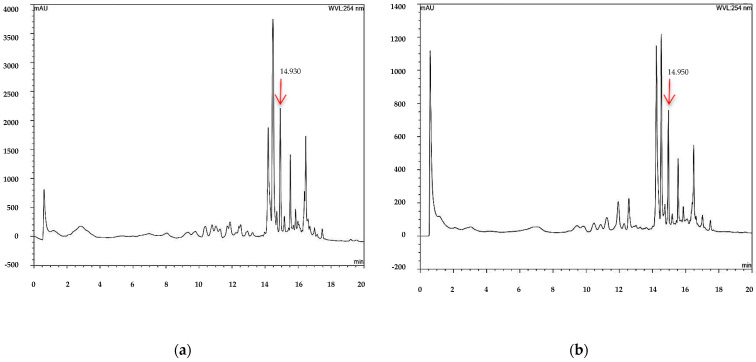
Chromatogram of the acetone extract of aerial parts of *Heteroteca inuloides,* sample 1 (**a**) and sample 2 (**b**).

**Figure 3:**
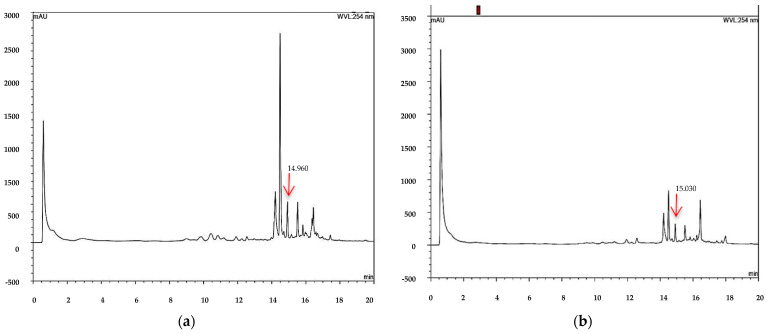
Chromatogram of the methanol extract of aerial parts of *Heteroteca inuloides,* sample 1 (**a**) and sample 2 (**b**).

The injection of a standard solution of 7-hydroxy-3,4-dihydrocadalene showed only a signal peak at 14.99 min (Figure 1). Subsequently, when performing the identification in the acetonic extracts of samples 1 and 2 (Figure 2), the same signal is observed at 14.930 and 14.950 min, indicating the presence of 7-hydroxy-3,4-dihydrocadalene in both extracts. In the same way, the presence of the compound was identified (Figure 3) in the methanolic extracts of samples 1 and 2, since a peak is shown at 15.030 and 14.960 min, with those results it is clear that the proposed method was adequate to identify 7-hydroxy-3,4-dihydrocadalene and its quantification in acetone and methanolic extracts of aerial parts of *H. inuloides* is possible.

### 2.4. Insecticidal Activity of 7-Hydroxy-3,4-dihydrocadalene against Spodoptera frugiperda

The evaluation carried out with 7-hydroxy-3,4-dihydrocadalene and *S. frugiperda* indicated that concentrations of 40, 60 and 100 ppm yielded significant responses in larval mortality, registering values of 60, 75 and 55%, respectively (Table 5). In the case of pupal mortality, no concentration yielded a significantly different response to the control. For cumulative mortality, it was observed that all concentrations gave significant responses, with values of 80, 75, 60 and 60% for 100, 60, 40 and 20 ppm, respectively. The larval LC_50_ was 44.36 ppm, and the cumulative LC_50_ was 29.41 ppm.

### 2.5. Ecotoxicological Activity of 7-Hydroxy-3,4-dihydrocadalene against Poecilia reticulata

Once the insecticidal activity on *S. frugiperda* of the major compound of the acetonic extract of *H. inuloides* was found and evaluated, its toxicological effect was evaluated on fingerlings, adult females, and males of *P. reticulata* (Table 6). A mortality rate of 100% was observed at a concentration of 10 ppm for fingerlings and male and female adults at 6 h of exposure for all the bioassays, showing a cumulative toxicity effect in the bioassays with fingerlings and female adults.

## 3. Discussion

### 3.1. Insecticidal Activity

Biological activity against *S. frugiperda* has been reported for extracts from plants belonging to the family Asteraceae; an example is the work of Romo-Asunción et al. (2016) [17], who reported larval mortality rates of 100, 95, 90 and 52.5% at 5000, 2000, 1000 and 500 ppm, respectively, with the hexanic extract of *Senecio salignus*. Salinas-Sánchez et al. (2012) [18], evaluated a hexanic, acetonic and methanolic extract of *Tagetes erecta*, recording mortalities of 48, 60 and 72% at 500 ppm, respectively. Da Silva et al. (2017) [19], applied an ethanolic extract of *Baccharis dracunculifolia* and obtained a larval mortality rate of 2.5% at 250 ppm of the extract.

The results obtained with the *H. inuloides* extracts evaluated here were similar to those reported by Romo-Asunción et al. (2016) [17] and Salinas-Sánchez et al. (2012) [18]. However, in both cases, there was a greater response than with the *H. inuloides* extract, since in the first study, a mortality rate of 52.5% was reported, and in the second, there were rates of 48, 60 and 72%, all at 500 ppm, which exceeded 45%, the maximum mortality rate reached with the extracts evaluated in this study at 500 ppm. In contrast, the mortality rate of 2.5% reported by Da Silva et al. (2017) [19] is lower compared to the one reported herein and in the other studies mentioned, which indicates that it had a lower biological response.

Other Asteraceae have reported biological activity against other insects; for example, Rodríguez-Hernández and López-Pérez (2001) [20] reported 100% mortality against *Zabrotes subfasciatus* adults when applying *Senecio salignus* root powder at 1% (*w*/*w*). Neenah et al. (2015) [21] recorded LC_50_ values of 37.1, 66.3 and 123.2 ppm when evaluating the essential oils of *Ageratum conyzoides*, *Achillea fragrantissima* and *Tagetes minuta* against adults of *Callosobruchus maculatus*. This indicates that some plants of the family Asteraceae have insecticidal activity against certain pest insects.

The biological activity of different sesquiterpene compounds against *S. frugiperda* has been reported by several authors. In 2017, Sosa et al. [22], found insecticidal activity of eudesmans (sesquiterpene-type compounds), which were isolated from *P. sagittalis*, and they registered a percentage of inhibition of dietary intake of 85% compared to the control at 100 µg and 200 µg/g. Another work where compounds of this type were used was that of Miranda et al. (2022) [23], who, from the sesquiterpene lactones tagitinin A, tagitinin C and 1 β-methoxydiversifoline, recorded decreases in the duration of the larval phase of 1.2, 1.88 and 1.29 d, all at a concentration of 100 ppm. On the other hand, Measured et al. (2021) [24] evaluated the activity of several grindelans (diterpene compounds) against *S. frugiperda* and reported that one achieved a larval mortality of 100% at 100 ppm. With what was reported in the aforementioned works, it can be observed that 7-hydroxy-3,4-dihydrocadalene showed a better response than the first two works. At first, there was only intake inhibition, while 7-hydroxy-3,4-dihydrocadalene had an accumulative LC_50_ of 29.41 ppm. On the other hand, the results presented by Mesurado et al. (2022) [24], show a better response than those obtained in the present investigation, since in the referenced work, a mortality of 100% was reached at 100 ppm, a concentration that in this work I recorded a larval mortality of 55%.

### 3.2. Ecotoxicology Activity

The LC_50_ for each test was 12.39 ppm in fingerlings, 62.94 ppm in adult males and 103.67 ppm in adult females, classifying the extracts, according to the criteria of Helfrich et al. (2009) [25], as slightly toxic to fingerlings and adult males and minimally toxic to adult females. The TL_50_ in each trial was 4.5 h in fingerlings, 2.78 h in adult males, and 2.67 h in adult females.

A lower-concentration LC_50_ was observed in fingerlings than in adults (female and male), which is a consequence of the susceptibility to the absorption of xenobiotics in younger *P. reticulata* specimens. This can be attributed to the fact that the immature fish were more susceptible, as was reported by Connaughton (2015) [26].

The LC_50_ values were used to determine the degree of toxicity of the methanolic extracts from the aerial parts of *H. inuloides*. The bioassays in fingerlings, adult males and females (187.16, 223.97 and 153.74 ppm, respectively) of *P. reticulata* were classified within the range of minimally toxic according to the criteria of Helfrich et al. (2009) [25].

The LC_50_ values obtained in the present bioassays show that the acetonic extracts have lower toxicity values compared to the methanolic extracts (fingerlings MetOH: 187.16 ppm and Acet: 12.39 ppm; adult males MetOH: 223.97 ppm and Acet: 62.94 ppm; female adults MetOH: 153.74 ppm and Acet: 103.67 ppm). These results indicate that there is a lethal effect on 50% of the *P. reticulata* population at low concentrations of the acetonic extract compared to the methanolic extract.

Likewise, the LC_50_ found for the acetonic extract in the bioassays with fingerlings correspond to a toxicological effect at a lower concentration compared to the bioassays with female and male adults. This effect may be because the fingerlings do not have fully matured organs or tissues responsible for the transformation and elimination of toxic contaminants, such as the kidney, intestine, and liver.

The results obtained show an LC_50_ of 0.89 ppm in fingerlings, 4.09 ppm in male adults and 0.98 in female adults, values classify the compound as highly toxic (fingerlings and adult females) and moderately toxic (adult males). NOM-232-SSA1-2009 [27] classifies this pesticide as extremely toxic in fingerlings and female adults and moderately toxic in male adults. The TL_50_ in each trial was 0.96 h in fingerlings, 2 h in adult males, and 1.21 h in adult females.

During the bioassays, unusual behavior was identified in the fish minutes before their death, highlighting erratic swimming and immobility while staying at the bottom of the container. Likewise, the fish presented accelerated movement in their gills and an increase in swimming mainly in the bioassays with the highest concentration. These effects are similar to those reported for specimens of the same species during exposure to thymol [28].

The LC_50_ values found are similar to those reported with methyl parathion (8.48 ppm) and chlorpyrifos (0.176 ppm); both compounds are present in organophosphorus synthetic chemical pesticides. The authors also reported opercular movement, abnormal swimming, and hyperactivity in guppy fish prior to death, similar to the behaviors observed in the present work [29]. Additionally, deltamethrin, a synthetic pyrethroid chemical pesticide, presented an LC_50_ of 0.38 ppm in *Labeo rohita* carp [30].

The results obtained show a greater toxicity of the pyrethroid pesticide than of the acetonic and methanolic extracts of *H. inuloides* in specimens of *P. reticulata*, which may be due to a deficiency in the enzymatic system of fish that hydrolyzes [31].

The majority compound presented an octanol–water partition coefficient (log P) of 4.54 [32]. This indicates that it has a greater affinity for fatty tissues responding to its toxicity in guppy fish [33]. Its affinity for lipophilic compounds facilitates its adsorption in the gills and decreases its elimination when already adsorbed [34]. This type of compound is classified as a substance with a real bioconcentration potential due to having a log *p* value > 4 [35].

Botanical extracts and sesquiterpenes are not listed in NOM-0.52SEMARNAT-2005 [36] as dangerous substances. The present study demonstrates they should be included due to the moderate toxicity of 7-hydroxy-3,4-dihydrocadalene in guppy fish.

### 3.3. Identification of 7-Hydroxy-3,4-dihydrocadalene

7-hydroxy-3,4-dihydrocadalene is a bioactive compound of aerial parts of *H. Inuloides* acetone extract, and with increasing applications of botanical of extracts with insecticide activity, it is essential to determinate an analytical method for its identification. In this sense, Yan et al. [37] identified four cadinenes from Eupatorium adenophorum by HPLC, which were 9-oxo-10,11-dehydroageraphorone, muurol-4-en-3,8-dione, 9-oxo-ageraphorone, and 9b-hydroxy-ageraphorone, all of them with antifeedant activity against *Spodoptera exigua*. On the other hand, Giuccione et al. [38] proposed a rapid method to analyze sesquiterpene lactones costunolide and dehydrocostus lactone by HPLC from roots of *Aucklandia lappa*, Also, Chib et al. [39] proposed a HPLC method to identify and quantify parthenin and coronopilin from Parthenium hysterophorus. In this research, the HPLC analytical method was proposed, which can be used to identify the 7-hydroxy-3,4-dihydrocadalene in aerial parts of acetone extract of *H. inuloides*.

## 4. Materials and Methods

### 4.1. Obtaining the Extracts

Two 250 g samples of aerial parts of *H. inuloides* were collected at two sites in the municipality of Atlixco, Puebla, Mexico (both located between the geographic coordinates 18°49′30″, 18°58′30″ north latitude and 98°18′24″, 98°33′36″ west longitude). The plant material was authenticated by E. Linares and R. Bye, and one specimen was deposited in the Botanical Garden of the Institute of Biology of the National Autonomous University of Mexico (UNAM), with a voucher specimen number (R. Bye + E. Linares 19401).

The collected plant material was transferred to the Institute of Chemistry of UNAM, and the sample was dehydrated for 3 weeks under ambient conditions under shade and then ground in a mortar until a particle size of approximately 2 cm was obtained. Subsequently, extraction was carried out using acetone and methanol (reagent grade, J.T. Baker) as solvents in which plant material was placed inside a 2-L Erlenmeyer flask, maintaining the 1:5 ratio (plant material/solvent). The extraction was carried out by the maceration at room temperature (3 times/24 h). The supernatant of maceration was filtered, collected, and evaporated to dryness using an R II rotary evaporator (BÜCHI Labortechnik AG, Flawil, Switzerland).

### 4.2. Reproduction of Spodoptera frugiperda and Poecilia reticulata

#### 4.2.1. *Spodoptera frugiperda*

*Spodoptera frugiperda* larvae were obtained from a colony established in the Laboratory of Natural Insecticide Compounds of the Faculty of Chemistry of Autonomous University of Querétaro (FQ-UAQ). The insects were fed an artificial diet based on the work of Ramos-López et al. (2010) [40], as shown in Table 7. The bioassays were approved by the Bioethics Committee of FQ-UAQ (Official No.: CBQ21/073).

The diet was made from the addition of all the components listed in Table 7 in a 2 L container, with the exception of ascorbic acid, formaldehyde, agar, water and ethanol. The agar was added to 400 mL of water and brought to boiling temperature, and then the rest of the water was added. Immediately after mixing the agar with the water, it was incorporated into the container with the rest of the components, also adding the ascorbic acid dissolved in ethanol and the formaldehyde and letting the mixture cool for 30 min.

The larvae were individually placed in Primo no. 0 polyethylene cups (Ecatepec Edo., Mexico) together with a 1 cm^3^ piece of the artificial diet. They were kept in a bioclimatic chamber at conditions of 27 ± 2 °C, 70 ± 5% relative humidity, and a light/dark photoperiod of 14:10 h. Upon reaching the pupal stage, groups of 50 *S. frugiperda* pupae were placed in 1 L plastic containers and kept under the same conditions. After the adults emerged, they were placed in waxed paper bags in groups of 50 adults with a 2:1 female/male ratio to allow copulation and oviposition of egg masses on the bag walls. During this phase, the adults were placed in a 10% sucrose solution for feeding, and the bag was changed every third day.

#### 4.2.2. *Poecilia reticulata*

##### Ethical Approval for Animal Use

The experimental procedures and animal handling were carried out based on the 203 OECD standard (OECD, 1992) [41]. In addition, the bioassays carried out in this work were approved by the Bioethics Committee of FQ-UAQ (Official No.: CBQ19/023). The *Poecilia reticulata* individuals were obtained from the Laboratory of Chemical Analysis of Live Food, belonging to the Department of Man and his Environment of the Autonomous Metropolitan University Xochimilco Unit.

##### Acclimatization and Conditioning

The *P. reticulata* specimens were placed in a fish tank with 40 L of semihard water (Table 8), in which aquatic plants (*Ceratophyllum demersum*, Ceratophyllaceae) were added to reduce stress on the fish. In addition, a filter (Aquajet, Sevilla, Spain) and an aerator (Hagen, Baie-d’Urfé, Canada) were also added. The dissolved oxygen values were maintained at 5.1 mg L^−1^ (60.2%), the water temperature at 20.7 ± 1.2 °C and the photoperiod at 14:10 h light/dark under ambient conditions. Subsequently, 3 fish L^−1^ of water were placed in the tank [42,43] and fed ad libitum a diet of Tetra^®^ fish flakes (Germany).

Then, the dead fish were placed in yellow polyethylene bags with the biohazard symbol as indicated in the official Mexican standard NOM-087-SEMARNAT-SSA1-2002 [44]. The bags were closed and transferred to the temporary hazardous waste warehouse of the Faculty of Chemistry and later disposed of according to the private company with which the faculty has an agreement.

### 4.3. Biological Activity of the Acetonic and Methanolic Extracts of the Aerial Parts of Heterotheca inuloides on Spodoptera frugiperda and Poecilia reticulata

#### 4.3.1. *Spodoptera frugiperda* Bioassay

To evaluate the effects of different concentrations of the extracts, a preliminary test was carried out with logarithmic concentrations from 0.5 to 5000 ppm to observe the maximum and minimum biological responses. Each concentration were added during the artificial diet preparation, using polyvinylpyrrolidone (PVP) (Sigma-Aldrich, Toluca, Mexico) as a cosolvent and also was added in control. The final concentrations were determined, which were 0, 500, 1000, 2000 and 5000 ppm for the acetonic and methanolic extracts of sample 1 and 0, 100, 400, 600 and 1000 ppm for those of sample 2. A completely randomized experimental design was adopted, with 4 replicates with 5 experimental units (EUs) each. A second instar larva was placed in a Primo no. 0 cup with a 1 cm^3^ diet cube under the same conditions as the broodstock. The bioassay was reviewed every 24 h until the larvae reached the last larval stage. Once the sixth instar was reached, the dependent variables (larval mortality, pupal mortality, cumulative mortality, and LC_50_) were evaluated.

#### 4.3.2. *Poecilia reticulata* Bioassay

The evaluation of ecotoxicological activity was carried out in fingerlings (less than two days old), adult males (weight 0.168 g and length 2.8 cm) and adult females (weight 0.849 g and length 3.5 cm) of *P. reticulata*. The concentrations of the acetonic and methanolic extracts evaluated were 500, 250, 125, 62.5 and 31.2 mg L^−1^ in the case of adult males and females, while for the fingerlings, they were 100, 10, 1, 0.1 and 0.01 mg L^−1^. For each of the concentrations, three fishes of each phase were used for a total 9 EUs with three replicates each [45,46].

For the preparation of the concentrations of the acetone extract, semihard water prepared for fish rearing was used, in addition to polyvinylpyrrolidone (PVP) (reagent grade, J.T. Baker) as a cosolvent in a 1:2 extract/PVP ratio; therefore, for the control, this substance was present in water. All the bioassays were carried out at a temperature similar to that described in the acclimatization stage, but without aeration and without feeding during all the evaluations. Mortality was recorded at 1, 3, 6, 12, 24, 48, 72 and 96 h after exposure, and individuals were considered dead when no opercular movement was observed. With these data, the lethal concentration was calculated as the mean lethal concentration (LC_50_) and mean lethal time (TL_50_) [41].

In the case of the methanolic extract, no PVP cosolvent was used, so the control consisted of only water. The conditions during the evaluation as well as the mortality recording times were the same as those applied to the acetonic extract, and similarly, the LC_50_ and TL_50_ values were calculated.

### 4.4. Identification of the Major-Compound

The compound 7-hydroxy-3,4-dihydrocadalene was identified by high performance liquid chromatography (HPLC) using a Thermo Fisher Scientific Ultimate 3000 system with a binary pump, HL diode array detector and an Agilent 927975-902 ZORBAX Eclipse XDB-C18 column (4.6 × 50 mm, 1.8-µm particle size). The injected volume was 20 μL in triplicate with a run time of 20 min. For chromatographic analysis, 40 mg of extract were accurately weighed then transferred into a 10 mL calibrated flask and dissolved in 1 mL of HPLC-grade methanol. The solution was brought to a volume of 10 mL with methanol to give a final concentration equivalent to 4 mg/mL. The standard solution of 7-hydroxy-3,4-dihydrocadalene was prepared in methanol by weighing out 50 g of pure compound into 10 mL volumetric flask to have a final concentration of 500 µg mL^−1^. The solutions were filtered through a Millex-HN^®^ syringe filter with a 0.45 μm nylon hydrophilic membrane (Merck KGaA, Darmstadt, Germany) and solutions were then transferred into 5 mL glass vials. The HPLC instrument was equipped with a C18 reversed-phase analytical column of 50 mm × 4.6 mm and a 3.5 μm particle (Agilent Technologies, Santa Clara, CA, USA) was operated at 30 °C. The samples were analyzed by HPLC with a mobile phase composed of mixture of methanol (solvent A) and water containing 0.1% acetic acid (solvent B) using a gradient elution (from 50/50 to 100/0%, *v*/*v*) at a flow rate of 1.0 mL min^−1^, with 20 min total running time.

Data acquisition and peak integration were performed with Dionex™ Chromeleon™ Chromatography Data System software 6.80 SR15 Build 4635 (Beta) (Thermo Fisher Scientific Inc. Waltham, MA, USA).

A sample of 7-hydroxy-3,4-dihydrocadalene was donated by Dr. Guillermo Delgado, and the pure compound was obtained following the method described by Delgado et al. (2001) [47].

### 4.5. Effect of the Majority Compound on Spodoptera frugiperda and Poecilia reticulata

#### 4.5.1. *Spodoptera frugiperda* Bioassay

For the evaluation of 7-hydroxy-3,4-dihydrocadalene, the same procedure described for the evaluation of the extracts was followed, evaluating the concentrations of 0, 20, 40, 60 and 100 ppm. Similarly, the corresponding PROBIT analysis was performed to calculate the LC_50_.

#### 4.5.2. *Poecilia reticulata* Bioassay

Three bioassays were carried out with specimens of fingerlings, male adults and female adults, which were randomly selected; three specimens per EU and three replicates were used. The 7-hydroxy-3,4-dihydrocadalene was co-dissolved with PVP in a 1:2 compound/PVP ratio. Five concentrations (100, 10, 1, 0.1 and 0.01 mg L^−1^) were prepared using water as the solvent in 250-mL beakers; as a negative control, water with the highest concentration of PVP was used. The fish were subjected to the same temperature conditions as in the acclimatization stage, but without aeration and without feeding. The fish were checked every 1, 3, 6, 12, 24, 48, 72 and 96 h; the LC_50_ and TL_50_ were calculated.

### 4.6. Statistical Analysis

The results obtained in the insecticidal and ecotoxicological activity bioassays were subjected to one-way analysis of variance (ANOVA) followed by Tukey’s test, as well as PROBIT analysis to determine the median lethal concentration (LC_50_), and also the mean lethal time (TL_50_) were calculated for the ecotoxicological bioassay. Every statistical analyses were performed at 95% confidence using SYSTAT 9 statistical software.

## 5. Conclusions

Both the methanolic and acetonic extracts showed insecticidal activity against *S. frugiperda*, with a better response for the acetonic extracts.

The acetonic and methanolic extracts of *H. inuloides* present ecotoxicological activity against *P. reticulata* and were classified as slightly and minimally toxic, respectively.

The identification of 7-hydroxy-3,4-dihydrocadalene from the acetonic extract was achieved, and the compound showed good insecticidal activity against *S. frugiperda*; however, it was classified as highly toxic on adult males of *P. reticulata* and moderately toxic on fingerlings and adult females.

Extracts of *H. inuloides* can be used as botanical control agents for pest management of *S. frugiperda* due to their efficacy, but more studies are needed to reduce its toxicity to nontarget organisms, and also the quantification and standardization the acetone extract of the aerial parts of *H. inuloides* can be possible using this compound as a marker.

## Figures and Tables

**Table 1:** Insecticidal activity of the acetonic extract of sample 1 and 2 of *Heterotheca inuloides*.

**Acetonic Extract of Sample 1**			
**Treatment (ppm)**	**Larval Mortality (%)**	**Pupal Mortality (%)**	**Cumulative Mortality (%)**
5000	90.0 ± 6.9 ^A^	10.0 ± 6.9 ^A^	100.0 ± 0 ^A^
2000	70.0 ± 10.5 ^AB^	15.0 ± 8.2 ^A^	85.0 ± 8.2 ^AB^
1000	85.0 ± 8.2 ^A^	0 ± 0 ^A^	85.0 ± 8.2 ^AB^
500	45.0 ± 11.4 ^BC^	20.0 ± 9.2 ^A^	65.0 ± 10.9 ^B^
0	15.0 ± 8.2 ^C^	5.0 ± 5.0 ^A^	20.0 ± 9.2 ^C^
	*p* < 0.0001	*p* = 0.241	*p* < 0.0001
LC_50_	730.4 ppm		401.5 ppm
**Acetonic Extract of Sample 2**			
**Treatment (ppm)**	**Larval Mortality (%)**	**Pupal Mortality (%)**	**Cumulative Mortality (%)**
5000	90.0 ± 6.9 ^A^	10.0 ± 6.9 ^A^	100.0 ± 0 ^A^
2000	70.0 ± 10.5 ^AB^	15.0 ± 8.2 ^A^	85.0 ± 8.2 ^AB^
1000	85.0 ± 8.2 ^A^	0 ± 0 ^A^	85.0 ± 8.2 ^AB^
500	45.0 ± 11.4 ^BC^	20.0 ± 9.2 ^A^	65.0 ± 10.9 ^B^
0	15.0 ± 8.2 ^C^	5.0 ± 5.0 ^A^	20.0 ± 9.2 ^C^
	*p* < 0.0001	*p* = 0.241	*p* < 0.0001
LC_50_	717.7 ppm		609.8 ppm

Each value is the mean of 20 data points ± mean standard error. Treatments with different letters in the same column indicate statistical differences (ANOVA and Tukey *p* < 0.05).

**Table 2:** Insecticidal activity of the metanolic extract of sample 1 and 2 of *Heterotheca inuloides*.

**Metanolic Extract of Sample 1**			
**Treatment (ppm)**	**Larval Mortality (%)**	**Pupal Mortality (%)**	**Cumulative Mortality (%)**
5000	85.0 ± 8.19 ^A^	0 ± 0 ^B^	85.0 ± 8.19 ^A^
2000	40.0 ± 11.2 ^B^	35.0 ± 10.9 ^A^	75.0 ± 9.93 ^AB^
1000	35.0 ± 10.9 ^B^	35.0 ± 10.9 ^A^	70.0 ± 10.9 ^AB^
500	35.0 ± 10.9 ^B^	5.0 ± 5.0 ^B^	40.0 ± 11.2 ^BC^
0	10.0 ± 6.89 ^B^	5.0 ± 5.0 ^B^	15.0 ± 8.19 ^C^
	*p* < 0.0001	*p* = 0.001	*p* < 0.0001
LC_50_	2302.0 ppm		1033.8 ppm
**Metanolic Extract of Sample 2**			
**Treatment (ppm)**	**Larval Mortality (%)**	**Pupal Mortality (%)**	**Cumulative Mortality (%)**
5000	60.0 ± 11.2 ^A^	5.0 ± 5.0 ^A^	65.0 ±10.9 ^A^
2000	45.0 ± 11.4 ^AB^	10.0 ± 6.88 ^A^	55.0 ± 11.4 ^AB^
1000	30.0 ± 10.5 ^AB^	5.0 ± 5.0 ^A^	35.0 ± 10.9 ^ABC^
500	10.0 ± 6.88 ^B^	0 ± 0 ^A^	10.0 ± 6.88 ^BC^
0	10.0 ± 6.88 ^B^	10.0 ± 6.88 ^A^	0.0 ± 9.18 ^C^
	*p* < 0.001	*p* = 0.66	*p* < 0.001
LC_50_	781.3 ppm		681.3 ppm

Each value is the mean of 20 data points ± mean standard error. Treatments with different letters in the same column indicate statistical differences (ANOVA and Tukey *p* < 0.05).

**Table 3:** Toxicological activity (% mortality) of the acetone extract of the aerial parts of *Heteroteca inuloides* on (A) fingerlings, (B) male adults and (C) female adults of *Poecilia reticulata*.

**A. Fingerlings**										
**Treatment (ppm)**	**Time (Hours)**									
	**1**	**3**	**6**	**12**	**24**	**48**	**72**	**96**	**Total**	**TL_50_**
500	0	0	100	-	-	-	-	-	100.00 ^A^	4.49
250	0	0	0	0	11.11	0	0	0	11.11 ^B^	178.94
125	0	0	0	0	0	0	0	0	0.00 ^B^	-
62.5	0	0	0	0	0	0	0	0	0.00 ^B^	-
31.2	0	0	0	0	0	0	0	0	0.00 ^B^	-
0	0	0	0	0	0	0	0	0	0.00 ^B^	-
CL_50_	12.39 ppm									
**B. Male Adults**										
**Treatment** **(ppm)**	**Time (Hours)**									
	**1**	**3**	**6**	**12**	**24**	**48**	**72**	**96**	**Total**	**TL_50_**
500	100	-	-	-	-	-	-	-	100.00 ^A^	0.5
250	0	100	-	-	-	-	-	-	100.00 ^A^	2
125	0	77.78	22.22	-	-	-	-	-	100.00 ^A^	2.78
62.5	0	0	0	11.11	11.11	0	0	11.11	33.33 ^B^	117.23
31.2	0	0	0	0	0	0	0	22.22	22.22 ^B^	99.66
0	0	0	0	0	0	0	0	11.11	11.11 ^B^	-
CL_50_	62.94 ppm									
**C. Female Adults**										
**Treatment** **(ppm)**	**Time (Hours)**									
	**1**	**3**	**6**	**12**	**24**	**48**	**72**	**96**	**Total**	**LT_50_**
500	88.89	11.11	-	-	-	-	-	-	100.00 ^A^	0.5
250	0	88.89	11.11	-	-	-	-	-	100.00 ^A^	2.67
125	0	22.22	0	0	11.11	22.22	0	0	55.56 ^AB^	66.05
62.5	0	0	0	0	0	0	33.33	0	33.33 ^BC^	100.95
31.2	0	0	0	0	0	0	0	0	0.00 ^BC^	-
0	0	0	0	0	0	22.22	0	0	22.22 ^C^	-
LC_50_	103.67 ppm									

Each value is the result of the average of 9 data. LT_50_ = Lethal Time Fifty, LC_50_ = Lethal Concentration Fifty. Treatments with different letters in the same column indicate statistical differences (ANOVA and Tukey *p* < 0.05).

**Table 4:** Toxicological activity (% mortality) of the methanolic extract of the aerial parts of *Heteroteca inuloides* on (A) fingerlings, (B) male adults and (C) female adults of *P. reticulata*.

**A. Fingerling**										
**Treatment** **(ppm)**	**Time (Hours)**									
	**1**	**3**	**6**	**12**	**24**	**48**	**72**	**96**	**Total**	**TL_50_**
500	0	100	-	-	-	-	-	100	100.00 ^A^	2
250	0	0	0	44.44	22.22	22.22	11.11	0	100.00 ^A^	23.09
125	0	0	0	0	0	0	0	0	0.00 ^B^	-
62.5	0	0	0	0	0	0	0	0	0.00 ^B^	-
31.2	0	0	0	0	0	0	0	0	0.00 ^B^	-
0	0	0	0	0	0	0	0	0	0.00 ^B^	-
CL_50_	187.16 ppm									
**B. Male Adults**										
**Treatment** **(ppm)**	**Time (Hours)**									
	**1**	**3**	**6**	**12**	**24**	**48**	**72**	**96**	**Total**	**TL_50_**
500	11.11	88.89	-	-	-	-	-	-	100.00 ^A^	1.32
250	0	0	0	0	11.11	11.11	11.11	11.11	44.44 ^AB^	93.5
125	0	0	0	0	0	11.11	0	0	11.11 ^B^	151.87
62.5	0	0	0	0	11.11	11.11	0	0	22.22 ^B^	123.56
31.2	0	0	0	11.11	11.11	0	0	0	22.22 ^B^	138.7
0	0	0	0	0	0	11.11	11.11	0	22.22 ^B^	-
CL_50_	223.97 ppm									
**C. Female Adults**										
**Treatment** **(ppm)**	**Time (Hours)**									
	**1**	**3**	**6**	**12**	**24**	**48**	**72**	**96**	**Total**	**TL_50_**
500	88.89	11.11	-	-	-	-	-	-	100.00 ^A^	0.5
250	11.11	33.33	0	11.11	0	0	0	0	55.56 ^AB^	44.91
125	0	22.22	11.11	0	0	11.11	0	0	44.44 ^AB^	90.57
62.5	0	0	11.11	11.11	0	0	11.11	0	33.33 ^B^	117.25
31.2	11.11	11.11	11.11	0	0	0	0	0	33.33 ^B^	196.71
0	0	11.11	11.11	0	0	0	0	0	22.22 ^B^	-
CL_50_	153.74 ppm									

Each value is the result of the average of 9 data. LT_50_ = Lethal Time Fifty, LC_50_ = Lethal Concentration Fifty. Treatments with different letters in the same column indicate statistical differences (ANOVA and Tukey *p* < 0.05).

**Table 5:** Insecticidal activity of 7-hidroxi-3,4-dihidrocadalene.

Treatment (ppm)	Larval Mortality (%)	Pupal Mortality (%)	Mortality Accumulated (%)
100	55.0 ± 11.4 ^A^	25.0 ± 9.93 ^A^	80.0 ± 9.18 ^A^
60	75.0 ± 9.93 ^A^	0 ± 0 ^B^	75.0 ± 9.93 ^A^
40	60.0 ± 11.2 ^A^	0 ± 0 ^B^	60.0 ± 11.2 ^A^
20	50.0 ± 11.5 ^AB^	10.0 ± 6.8 ^AB^	60.0 ± 11.2 ^A^
0	10.0 ± 6.88 ^B^	5.0 ± 5.0 ^AB^	15.0 ± 8.19 ^B^
	*p* = 0.001	*p* = 0.018	*p* < 0.0001
LC_50_	44.36 ppm		29.41 ppm

Note: Each value is the result of the mean of 20 data ± mean standard error. Treatments with different letters in the same column indicate statistical differences (ANOVA and Tukey *p* < 0.05).

**Table 6:** Toxicological activity of 7-hydroxy-3.4-dihydrocadalene on (A) fingerlings, (B) male adults and (C) female adults of *Poecilia reticulata*.

**A. Fingerlings**										
**Treatment** **(ppm)**	**Time (Hours)**									
	**1**	**3**	**6**	**12**	**24**	**48**	**72**	**96**	**Total**	**TL_50_**
100	100	0	0	0	0	0	0	0	100.00 ^A^	0.5
10	55.56	44.44	0	0	0	0	0	0	100.00 ^A^	0.96
1	0	0	0	0	22.22	33.33	11.11	11.11	77.78 ^A^	59.7
0.1	0	0	0	0	0	0	0	0	0.00 ^B^	-
0.01	0	0	0	0	0	0	0	0	0.00 ^B^	-
0	0	0	0	0	0	0	0	0	0.00 ^B^	-
CL_50_	0.89 ppm									
**B. Male Adults**										
**Treatment** **(ppm)**	**Time (Hours)**									
	**1**	**3**	**6**	**12**	**24**	**48**	**72**	**96**	**Total**	**TL_50_**
100	100	-	-	-	-	-	-	-	100.00 ^A^	0.5
10	0	100	-	-	-	-	-	-	100.00 ^A^	2
1	0	0	0	0	0	0	0	0	0.00 ^B^	-
0.1	0	0	0	0	0	0	0	0	0.00 ^B^	-
0.01	0	0	0	0	0	0	0	0	0.00 ^B^	-
0	0	0	0	0	11.11	11.11	0	0	22.22 ^B^	-
CL_50_	4.09 ppm									
**C. Female Adults**										
**Treatment** **(ppm)**	**Time (Hours)**									
	**1**	**3**	**6**	**12**	**24**	**48**	**72**	**96**	**Total**	**TL_50_**
100	100	-	-	-	-	-	-	-	100.00 ^A^	0.5
10	22.22	77.78	-	-	-	-	-	-	100.00 ^A^	1.21
1	0	11.11	0	0	22.22	0	0	22.22	55.56 ^B^	86.07
0.1	0	0	0	0	0	0	0	0	0.00 ^C^	-
0.01	0	0	0	0	0	0	0	0	0.00 ^C^	-
0	0	0	0	0	0	0	0	0	0.00 ^C^	-
CL_50_	0.98 ppm									

Each value is the result of the average of 9 data. LT_50_ = Lethal Time Fifty, LC50 = Lethal Concentration Fifty, Treatments with different letters in the same column indicate statistical differences (ANOVA and Tukey *p* < 0.05).

**Table 7:** Ingredients for the preparation of 1 kg of artificial diet to feed *Spodoptera frugiperda*.

Substance	Quantity
Ground corn	120.0 g
Ground beans	60.0 g
Freeze dried yeast	20.0 g
Neomicine	0.6 g
Multivitamin	2.5 g
Ascorbic acid	1.7 g
Metil 4-hidroxibenzoate	1.7 g
Bacteriological agar	10.0 g
Formaldehyde (10%)	2.5 mL
Water	800.0 mL
* Ethanol	17.0 mL

* It is used to dissolve ascorbic acid.

**Table 8:** Components of the reconstituted water for the acclimatization of *Poecilia reticulata*.

Substance	Formula	Quantity to Bring to 1 L of Distilled Water
Calcium chloride	CaCl_2_·2H_2_O	0.2940 g
Magnesium sulphate	MgSO_4_·7H_2_O	0.1233 g
Sodium bicarbonate	NaHCO_3_	0.0648 g
Potassium chloride	KCl	0.0058 g

Organization for Economic Cooperation and Development. *Fish, Acute Toxicity Test. Guideline for the Testing of Chemicals* [41].

## Data Availability

Not applicable.
